# Death Receptor Interactions With the Mitochondrial Cell Death Pathway During Immune Cell-, Drug- and Toxin-Induced Liver Damage

**DOI:** 10.3389/fcell.2019.00072

**Published:** 2019-04-24

**Authors:** Valentina Spinnenhirn, Janine Demgenski, Thomas Brunner

**Affiliations:** Biochemical Pharmacology, Department of Biology, University of Konstanz, Konstanz, Germany

**Keywords:** death receptor, TRAIL, DILI, Bcl2 family, Bim, JNK, APAP

## Abstract

Due to its extensive vascularization and physiological function as a filter and storage organ, the liver is constantly exposed to infectious and tumorigenic threat, as well as damaging actions of xenobiotics. Detoxification reactions are essential for the excretion of harmful substances, but harbor also the risk of “side effects” leading to dangerous metabolites of otherwise harmless substances, a well known effect during paracetamol overdose. These drugs can have detrimental effects, which often involves the induction of sterile inflammation and activation of the immune system. Therefore, the role of certain immune cells and their effector molecules in the regulation of drug-induced liver damage are of special interest. Hepatocytes are type II cells, and death receptor (DR)-induced cell death (CD) requires amplification via the mitochondrial pathway. However, this important role of the mitochondria and associated CD-regulating signaling complexes appears to be not restricted to DR signaling, but to extend to drug-induced activation of mitochondrial CD pathways. We here discuss the role of members of the TNF family, with a focus on TRAIL, and their interactions with the Bcl-2 family in the crosstalk between the extrinsic and intrinsic CD pathway during xenobiotic-induced liver damage.

## Liver Damage by Xenobiotics

**FIGURE 1:**
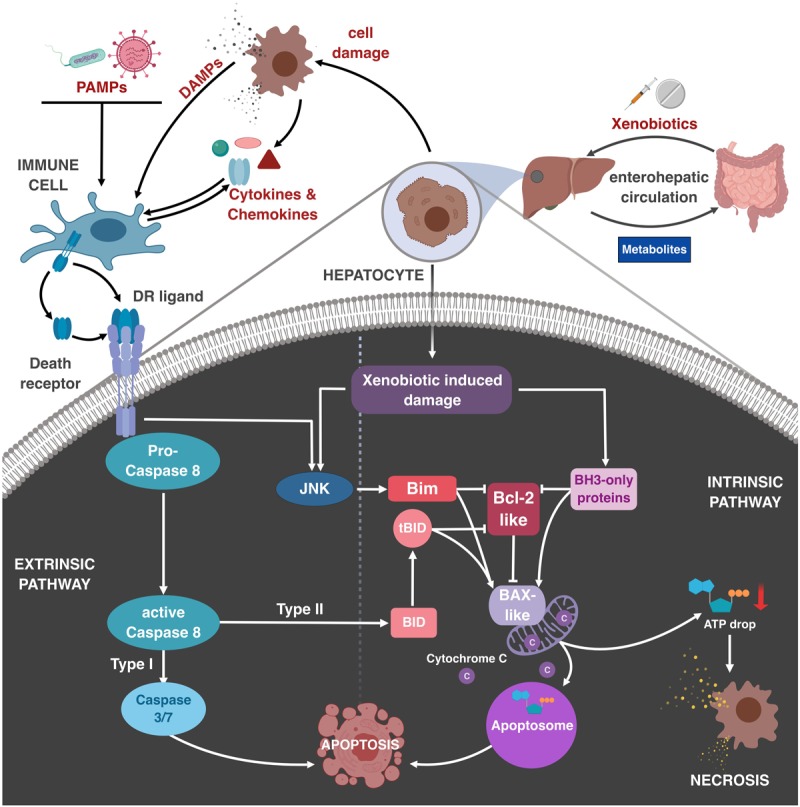
Role of immune cells and TNF family members in regulating drug-induced liver damage. Xenobiotics are absorbed in the gut and transported to the liver, where they become metabolized. During the metabolization, toxic intermediates may be generated, inducing damage to liver parenchymal cells, most importantly hepatocytes, via activation of the Bcl-2-regulated mitochondrial apoptosis pathway. This can lead to apoptosis via permeabilization of the outer mitochondrial membrane, cytC release and subsequent apoptosome formation, or necrotic CD if ATP levels are too low. Resulting DAMP (danger–associated molecular pattern) release by necrosis or late apoptosis can initiate sterile inflammation by activation of liver-resident immune cells, e.g., KC. DAMPs and PAMPs can stimulate immune cells, resulting in the release of effector molecules, e.g., cytokines and chemokines, which recruit and activate other immune cells. Additionally, DR ligands induce the extrinsic CD pathway. In hepatocytes already affected by xenobiotics DR activation promotes synergistic CD at otherwise sublethal concentrations. This crosstalk between DR and xenobiotics involves caspase-8-mediated cleavage of Bid, DR-induced activation of JNK and Bim, and xenobiotic-induced induction and activation of other BH3-only proteins. Resulting neutralization of pro-survival Bcl-2-like and activation of Bax-like molecules results in mitochondrial apoptosis or necrosis.

One major task of the liver is to maintain metabolic homeostasis. It processes and stores nutrients absorbed in the gut and delivered by the portal vein. In addition, as part of the enterohepatic circulation, the liver is the first organ to receive absorbed xenobiotics and toxins. Therefore, it provides a plethora of biochemical tools to metabolize, activate or inactivate drugs and poisons. Mainly involved are enzymes of the cytochrome P450 (CYP) family, which introduce functional groups. The subsequent conjugation and detoxification reactions enable the secretion of harmful chemicals via bile and kidney. Additionally, CYP enzymes are also involved in the chemical activation of inactive pro-drugs (e.g., cortisone or prednisone). These pharmacologically important reactions, called first-pass effect, are important for the regulation of activity and dosage of manydrugs. Next to beneficial activation and detoxification processes, these enzymatic reactions may also result in a detrimental outcome, e.g., via the chemical activation of otherwise harmless compounds, thereby gaining substantial toxic potential. Examples are N-acetyl-p-benzochinonimin (NAPQI), a reactive metabolite of acetaminophen, or aflatoxin B1 (AFB1). These reactive metabolites rather affect centrilobular hepatocytes, which have high CYP enzyme activity. Besides such indirect “side effects” of detoxification reactions, other chemicals may also directly induce liver toxicity. Such drugs or poisons can affect parenchymal and non-parenchymal cells of the liver, promoting a typically early onset of disease within a few days. Especially, hepatocytes in periportal regions of the liver lobules as well as endothelial cells are exposed to high levels of such xenobiotics. Altogether, these different forms of drug-induced liver injury (DILI) account for 50% of all cases with acute liver failure. Examples of medically relevant substances are acetaminophen (APAP, paracetamol), environmental toxins (AFB1), alcohol, carbon tetrachloride (CCl_4_), as well as antineoplastic agents. However, the bulk of DILI cases is attributed to APAP overdose. Generally, liver damage by xenobiotics can be induced by protein adducts and dysfunction, lipid peroxidation, DNA damage and glutathione depletion due to increased reactive oxygen species (ROS) levels, thereby inducing mitochondrial damage and impaired energy supply. Subsequent lytic necrosis and/or apoptosis can promote the release of cellular content, and associated induction of immune cell activation and sterile inflammation (Figure 1 upper part).

## The Role of the Immune System in Liver Damage

Liver-resident immune cells are important in the protection from enteric and liver-specific infections, as well as immune surveillance of liver metastases. Deregulated host immune reactions, however, are also a frequent cause of severe hepatitis. Similarly, metabolic disorders can induce detrimental liver damage and thereby activate effector mechanisms of the host immune system, e.g., as seen during metabolic syndrome, non-alcoholic fatty liver disease (NAFLD), non-alcoholic steatohepatitis (NASH), and alcoholic liver disease (ALD).

In this context also DILI is not necessarily only established by direct action of the respective compound on liver cells, but may involve a secondary response of the immune system. Important mediators during initiation and deregulation of such sterile inflammatory processes are so-called danger-associated molecular patterns (DAMPs). These normally intracellular molecules are typically released during necrotic CD due to loss of membrane integrity, but the process is likewise also relevant for apoptotic CD, though to a lesser extent. Immune cells are thus capable to distinguish “self-safe” and “self-dangerous” by evolutionary conserved pattern recognition receptors. Multiple transmembrane and intracellular receptors can sense DAMPs. They activate preferentially myeloid cells, resulting in the secretion of pro-inflammatory cytokines, like TNF and IFNγ, the recruitment of innate immune cells and further damage of the affected tissue. The vicious cycle of massive hepatocyte damage and extensive DAMP release is a well-known feed-forward loop interconnecting inflammation and CD during liver damage.

Kupffer cells (KC) are liver-resident tissue macrophages and the most abundant innate immune cells of the liver. Therefore, depletion of these phagocytic cells can have protective effects (Zhao et al., 2008; Kiso et al., 2012) but also exacerbate drug-induced liver damage due to their additional anti-inflammatory and tissue-protecting functions (Bourdi et al., 2002). Importantly, studies in human patients also suggest a role for infiltrating mononuclear cells in tissue repair processes, rather than promoting tissue damage (Antoniades et al., 2012). Natural killer cells (NK) and NKT cells are likewise part of the liver’s innate immune defense. Though liver-resident cells, they accumulate as infiltrates after initial liver damage. For APAP-induced DILI it was reported that NK and NKT cells play a disease-promoting role since increased NKT cell infiltrates and effector molecules, such as IFNγ, were observed. Additionally, NK and NKT depletion had a protective effect in APAP-induced DILI in mice (Liu et al., 2004). Though, this claim was challenged by others, and reported to be caused by side-effects of the solvent DMSO (Masson et al., 2008).

Independent of direct liver damage are immune reactions of the adaptive immune system, which are implicated in so-called unpredictable reactions, called idiosyncratic DILI (IDILI). These destructive mechanisms are not well understood, but are generally believed to be based on immune-mediated hypersensitivity (Schnyder et al., 1997; Chen et al., 2018).

## Immune Cell-Derived Death Ligands Affecting Drug-Induced Hepatitis

Drug- and toxin-induced hepatic insults, either by direct toxicity or caused by the adaptive immune system, are associated with deregulated inflammatory responses mediated by members of the TNF superfamily. This family comprises a multitude of membranous and soluble molecules. A subset of this family, including TNF, Fas ligand (FasL, CD95L) and TRAIL can activate so-called DRs and thereby the extrinsic CD pathway. Generally, sensitivity of CD induction by members of the TNF superfamily is highly regulated.

The most detrimental effect on hepatocytes is mediated by FasL, which is involved in various forms of immune cell-mediated acute liver damage. Activation of the Fas receptor on e.g., virus-infected or transformed hepatocytes leads to their rapid death. Released cellular content can modulate cells in close proximity to increase their FasL susceptibility, thereby further increasing bystander killing. Due to the abundant expression of Fas throughout the liver, systemic administration of FasL or agonistic anti-Fas antibodies results in acute and mostly fatal hepatitis (Ogasawara et al., 1993). Consequently, FasL expression and Fas-induced hepatocyte death need to be tightly controlled, e.g., by transcriptional control, post-translational regulation like intracellular storage and activation-dependent mobilization, and shedding by metalloproteases (Brunner et al., 2003). Functionally, FasL-induced apoptosis serves as a key effector mechanism in T- and NK cell-mediated cytotoxicity against Fas-expressing target cells.

Tumor Necrosis Factor plays a prominent role in the context of KC activation and associated liver pathology. It causes the induction of pro-inflammatory cytokines and chemokines, which promote recruitment of other inflammatory cells, induction of hepatocyte death or proliferation for wound healing responses. These pleiotropic effects on different cell types highlight it’s complex signaling output. In the healthy liver, for example, TNF produces non-apoptotic signals via activation of the MAP kinases JNK or p38, or NFκB, thereby triggering pro-survival and pro-inflammatory responses. Bifurcation of the signaling pathway is eventually regulated in a context-dependent manner. Thus, hepatocytes are rather insensitive to TNF-induced CD when administered alone. However, in combination with the liver-specific transcriptional inhibitor D-(+)-galactosamine (GalN) it causes massive CD. In this situation, the transcription-dependent pro-survival signaling of TNF cannot restrict the secondary activation of CD pathways, i.e., apoptosis or necroptosis.

Immediately after its discovery, the TNF homolog TRAIL gained extensive clinical interest due to its rather tumor-selective CD-inducing activity, even though this was also stated for untransformed cells. In the liver, TRAIL was reported to be involved in immune surveillance of tumors and metastases, but also in the control of viral infections. TRAIL is predominantly expressed by liver NK cells, where it contributes to their cytotoxic effector mechanisms together with perforin and FasL (Kayagaki et al., 1999; Takeda et al., 2001). TRAIL expression is regulated by IFNγ, which triggers TRAIL expression not only in NK cells, but also in monocytes and dendritic cells (DCs). Autocrine IFNγ production has been reported to promote constitutive TRAIL expression by liver-resident NK cells (Takeda et al., 2001). Receptor expression is, however, not sufficient to induce apoptosis by TRAIL, as the ratio of activating and decoy receptors, as well as anti-apoptotic proteins define sensitivity (Sarhan et al., 2014). Sensitivity might also be regulated by the crosstalk with the intrinsic apoptosis pathway. Many drugs that induce ER-stress, DNA damage or ROS sensitize otherwise TRAIL-resistant cells, leading to synergistic CD induction (Ganten et al., 2005; Koschny et al., 2007; Schneider-Jakob et al., 2010; Badmann et al., 2011).

### Synergy Between Death Ligands and Xenobiotics in Liver Toxicity

In contrast to FasL, TNF, and TRAIL may not only directly trigger the extrinsic CD pathway, but can also stimulate signaling pathways, which modify apoptosis initiated by other triggers. The cellular context seems to be especially relevant in this crosstalk between DR signaling and the intrinsic apoptosis pathway. A well-described player is the Bcl-2 family member Bid. In so-called type I cells, caspase activation after DR activation is sufficient to directly cause apoptosis. Type II cells, though, rely on the amplification of the extrinsic signal via the mitochondrial pathway (Yin et al., 1999). The best-characterized type II cells are hepatocytes. Low level of caspase-8 activation upon DR activation promotes cleavage of Bid, and its truncated form (tBid) mediates MOMP by direct activation of the pore-forming Bcl-2 family member Bax and neutralization of anti-apoptotic Bcl-2 homologs (Li et al., 1998). Besides this Bid-mediated crosstalk, other connections between different DRs and stress signaling pathways have added further levels of complexity. Initially, a crosstalk between TRAIL and the intrinsic pathway has been described in thymocytes. Thus, it was observed that TRAIL-deficiency results in reduced activation-induced thymocyte apoptosis upon T cell receptor (TCR) crosslinking, which is Bim-dependent (Corazza et al., 2004; Kassahn et al., 2008). Subsequently it was found that TRAIL enhances not only TCR-mediated apoptosis, but extends to other apoptosis triggers, like UV- and γ-irradiation, and glucocorticoids, but not Fas crosslinking. Given that thymocytes are type I cells, these findings suggested that TRAIL may specifically enhance the mitochondrial CD pathway. As in type II cells the Fas pathway is also amplified via mitochondria, it was tempting to speculate that TRAIL would also enhance Fas-induced hepatocyte apoptosis and associated liver damage. Indeed, it was found that Fas-induced hepatocyte apoptosis could be synergistically enhanced by TRAIL receptor activation (Corazza et al., 2006). Interestingly, this CD amplifying pathway initiated by TRAIL did not seem to depend on direct caspase activation, but rather on TRAIL receptor-initiated JNK activation and associated Bim phosphorylation. Consequently, TRAIL- and Bim-deficient mice, as well as mice treated with JNK inhibitors, were protected from anti-Fas-induced acute liver damage. Interestingly, similar observations have also been made for TNF. Kaufmann et al., 2009 described that both, Bid and Bim contribute to TNF-dependent LPS/GalN-induced liver damage (Kaufmann et al., 2009), whereas other publications observed a similar enhancing effect of TNF on Fas-induced hepatocyte apoptosis and associated liver damage, which was dependent on JNK and Bim (Schmich et al., 2011; Faletti et al., 2018). Thus, while lacking a direct hepatotoxic activity both TRAIL and TNF can activate the JNK-Bim axis and thereby enhance Fas-induced apoptosis in type II cells (Figure 1 lower left part).

Tumor Necrosis Factor-Related Apoptosis-Inducing Ligand, which induces CD in a p53-independent manner, has been regarded as an exciting alternative to conventional p53-dependent chemotherapy (Hellwig and Rehm, 2012). Various studies in different types of tumors revealed additive or even synergistic CD induction when TRAIL was combined with chemotherapy. Given the CD-enhancing effect of TRAIL in hepatocytes via activating the JNK-Bim axis, an obvious idea was that TRAIL would similarly regulate chemotherapy-induced apoptosis in tumor cells. Indeed, in hepatocellular carcinoma cells TRAIL stimulation results in JNK activation and Bim hyperphosphorylation (Schneider-Jakob et al., 2010). Furthermore, while tumor cells were relatively insensitive to TRAIL and chemotherapeutic drugs, a profound synergistic CD induction was seen when used in combination. This synergistic CD induction was strongly attenuated upon either pharmacological inhibition of JNK, or knockdown of Bim and Bid, confirming that TRAIL receptor-mediated activation of the JNK-Bim axis, likely together with the activation or transcriptional induction of other BH3-only proteins, represents the molecular basis for this synergy. Likely synchronized TRAIL- and chemotherapy-induced changes in the Bcl-2 interactome, ultimately resulting in efficient activation of Bax and Bak, and MOMP, are key events (Hantusch et al., 2018). TRAIL-induced and JNK-mediated phosphorylation of Bim appears to be an important switch in this process. It has been previously shown that Bim_L_ and Bim_EL_ interact with dynein light chain 1 (DLC1). While it was previously thought that DCL1 sequesters Bim at the cytoskeleton (Puthalakath et al., 1999), thereby inhibiting its apoptosis-inducing activity, more recent data shows that DLC1 is important for oligomerization of Bim and clustering it in high-molecular-weight complexes together with Mcl-1 at the mitochondrial outer membrane (Singh et al., 2017). Interestingly, the DLC1-binding domain of Bim overlaps with the phosphorylation site of JNK (T112). Thus, JNK-mediated phosphorylation appears to release Bim from this complex and thereby unleash its apoptosis-inducing activity by initiating Bax activation and neutralization of anti-apoptotic Bcl-2 homologs, such as Bcl-x_L_. Thereby it limits its Bax retrotranslocating activity, which seems to be critical for controlling Bax oligomerization and MOMP (Todt et al., 2015; Hantusch et al., 2018). Further adding to the level of complexity is the fact that some of the JNK-mediated phopsphorylation sites in Bim are overlapping with those of ERK1/2 (Lei and Davis, 2003; Ley et al., 2003). Yet, while JNK appears to have in general an activating activity on Bim, ERK1/2-mediated phosphorylation rather leads to Bim degradation and survival. Synergistic induction of CD in tumor cells by TRAIL and chemotherapy may represent promising strategies to overcome therapy resistance in tumor patient. However, it is worth mentioning that the mechanism *per se* seems to be far more general, as also primary human hepatocytes are sensitized by TRAIL to chemotherapeutic drug-induced apoptosis (Schneider-Jakob et al., 2010).

### Role of the TRAIL-JNK-Bim Axis in Enhancing Drug-Induced Liver Necrosis

As discussed above, APAP overdose is responsible for the vast majority of DILI cases. Interestingly, APAP overdose leads to necrotic lesions, rather than apoptotic liver CD. In addition, RIP1 and probably also RIP3 deficiency rescues from APAP toxicity, implicating a necrotic or necroptotic form of CD (Ramachandran et al., 2013; Dara et al., 2015). However, since MLKL inhibition has no beneficial effect on APAP pathology, necrosis remains to be the most relevant pathway. At the same time, APAP toxicity includes upstream apoptotic signaling events, like induction of pro-apoptotic Bcl-2 homologs, Bax activation and MOMP with release of cytC and Smac/DIABLO. Surprisingly, at least *in vivo* no caspase activation is seen, and caspase inhibitors do not prevent APAP-induced liver damage (Jaeschke et al., 2006). The question remains why caspases are not activated despite the extensive activation of the mitochondrial apoptosis pathway. It is well known that APAP treatment causes mitochondrial impairment and associated drop in ATP levels (Jaeschke, 1990). Furthermore, low ATP levels prevent apoptosome formation and caspase activation, shifting the CD execution toward necrosis (Nicotera et al., 1998). Therefore, it was suggested that APAP-induced decrease in ATP levels is responsible for shifting apoptotic processes toward a necrotic outcome. Indeed, preventing APAP-induced mitochondrial permeability transition by cyclosporine A, or increasing intracellular ATP by providing the glycolytic substrate fructose increases APAP-induced caspase activation in hepatocytes (Kon et al., 2004). Thus, current knowledge indicates that APAP-induced liver damage represents an interplay of several distinct CD mechanisms, including the activation of Bcl-2-family members and induction of MOMP, yet resulting in a necrotic outcome. Despite the lack of evidence for apoptosis induction, a role of certain Bcl-2 family members in the regulation of APAP-induced liver toxicity is well documented. Most importantly, the TRAIL-JNK-Bim axis seems to play also an important role in APAP-induced liver necrosis. Astonishingly, TRAIL or Bim deletion not only resulted in reduced APAP-induced hepatocyte death (Badmann et al., 2011), but also reduced death of liver sinusoidal endothelial cells (LSEC) (Badmann et al., 2012). Similarly, a profound role of JNK in the transcriptional upregulation of Bim, and the subsequent phosphorylation of Bim was observed (Badmann et al., 2011). These results clearly demonstrate that CD amplification via the TRAIL-JNK-Bim axis goes far beyond Bcl-2 family-regulated apoptosis induction via the mitochondrial pathway, but extends to necrotic form of liver CD and is likely not limited to APAP-induced liver damage. The question, however, remains how TRAIL can enhance hepatocyte necrosis. Does TRAIL indeed amplify APAP-induced necrosis and if so how? Or does it shift the cellular response from necrosis to apoptosis, which would likely involve a stabilization of intracellular ATP levels and apoptosome formation? And finally, how are these processes regulated by the Bcl-2 family members and their interactions? Especially in LSECs it could be shown that APAP and TRAIL synergistically induce apoptotic events followed by substantial caspase activation, which could indeed be rescued by the pan-caspase inhibitor zVAD (Badmann et al., 2012; Figure 1 lower right part). Mechanistically, the role of TRAIL and APAP in transcriptional and post-translational activation of Bim and other BH3-family members certainly has to be further addressed. In addition, the role of TRAIL in switching the APAP-induced CD pathway toward necrosis or apoptosis likewise remains an unsolved open question. Similarly, it remains to be investigated whether also other forms of DILI are regulated by TRAIL and the Bcl-2 family.

## Conclusion

Initial hepatocyte CD and subsequent liver damage induced by deregulated immune reactions are the common denominator of many severe forms of acute and chronic liver pathologies. However, the exact sequence of certain events, like e.g., the initial hepatotoxic insult, involvement of infiltrating and resident immune cells, cytokines, and secondary immune reactions, still need further clarification. Especially in the case of most immune cell-derived factors it needs to be carefully addressed whether they are cause or consequence of on-going CD events. In this regard, the role of death ligands in disease progression is of special interest, since they may add an additional complexity to the picture. Particularly the role of TRAIL, but also other modulating factors of intrinsic apoptosis pathways in liver parenchymal and endothelial cells, have not been taken into account by most *in vitro* studies. They need to be carefully interpreted since they do not reflect the *in vivo* situation where e.g., TRAIL is readily present by infiltrating, and most importantly resident, immune cells to modify xenobiotic- and drug-induced hepatotoxicity. The identification of the relevant underlying mechanisms of DILI, either mediated by apoptosis or necrosis, may help to develop effective emergency treatments to prevent drug-induced liver failure.

## Author Contributions

All authors drafted the manuscript. JD generated the illustration. VS and TB wrote the manuscript.

## Conflict of Interest Statement

The authors declare that the research was conducted in the absence of any commercial or financial relationships that could be construed as a potential conflict of interest. The reviewer UM declared a past co-authorship with one of the authors TB to the handling Editor.

## Abbreviations

APAP: acetaminophen, paracetamol
CD: cell death
cytC: cytochrome C
DAMP: danger associated molecular pattern
DILI: drug induced liver injury
DR: death receptor
JNK: c-Jun N-terminal kinase
KC: Kupffer cells
MOMP: mitochondrial outer membrane permeabilization
PAMPs: pathogen-associated molecular pattern
TNF: Tumor Necrosis Factor
TRAIL: Tumor Necrosis Factor-Related Apoptosis-Inducing Ligand
